# The Public’s Perception of the Severity and Global Impact at the Start of the SARS-CoV-2 Pandemic: A Crowdsourcing-Based Cross-Sectional Analysis

**DOI:** 10.2196/19768

**Published:** 2020-11-26

**Authors:** Orr Shauly, Gregory Stone, Daniel Gould

**Affiliations:** 1 Keck School of Medicine University of Southern California Los Angeles, CA United States

**Keywords:** Amazon Mechanical Turk, crowdsourcing, COVID-19, SARS-CoV-2, pandemic, perception, public opinion, survey, severity, impact, behavior, education

## Abstract

**Background:**

COVID-19 is a rapidly developing threat to most people in the United States and abroad. The behaviors of the public are important to understand, as they may have a tremendous impact on the course of this novel coronavirus pandemic.

**Objective:**

This study intends to assess the US population’s perception and knowledge of the virus as a threat and the behaviors of the general population in response.

**Methods:**

A prospective cross-sectional study was conducted with random volunteers recruited through Amazon Mechanical Turk, an internet crowdsourcing service, on March 24, 2020.

**Results:**

A total of 969 participants met the inclusion criteria. It was found that the perceived severity of the COVID-19 pandemic significantly differed between age groups (*P*<.001) and men and women (*P*<.001). A majority of study participants were actively adhering to the Centers for Disease Control and Prevention guidelines.

**Conclusions:**

Though many participants identified COVID-19 as a threat, many failed to place themselves appropriately in the correct categories with respect to risk. This may indicate a need for additional public education for appropriately defining the risk of this novel pandemic.

## Introduction

A novel coronavirus, COVID-19, has resulted in an ongoing global pandemic marked by a viral pneumonia with severe morbidity and mortality in 3%-5% of those infected [1,2]. With cases mounting in the United States over the past month and with the Centers for Disease Control and Prevention (CDC) and many local governments issuing safety measures including social distancing and cleanliness guidelines, the public has had to adapt to a new way of life [3,4]. However, these drastic changes may have a tremendous psychosocial impact.

The public’s perception of this pandemic’s severity may also impact adherence to CDC guidelines or regionally mandated quarantine, and suboptimal adherence to guidelines may have a detrimental effect on attempts to curb the continued spread of the COVID-19 pandemic. It is well known across many chronic health conditions (asthma or cardiovascular disease) and screening health tools such as colonoscopy and mammography that patient’s perception of severity or risk may directly correlate with adherence to treatment and guidelines [5-9]. It is thus important to survey the public’s perception of the pandemic’s severity and its psychosocial impact across age, sex, and individual risk factors.

## Methods

### Cross-Sectional Study

A prospective cross-sectional study of random volunteers recruited through an internet crowdsourcing service, Amazon Mechanical Turk (MTurk), was conducted. Study participants were recruited and performed the survey on March 24, 2020. Individuals were required to be older than 18 years and registered through the Amazon service platform within the United States to prevent workers from taking the same survey multiple times or from outside the United States, and the survey was not advertised. Internet crowdsourcing is a powerful tool in its ability to quantify the perceptions and opinions of a diverse group of individuals that may otherwise be inaccessible through other surveying methodologies at a fraction of the cost [10-13]. Several studies have shown that the participant population is extremely representative of the United States internet population, with 70%-80% of all workers originating and recording responses from the continental United States [11,14]. In this study, 100% of the workers were from the United States, as an Internet Protocol filter and registration filter were used to guarantee US response. Workers were provided with a compensation of US $0.25 per response (limit one per worker) and were screened by the platform for attentiveness to detail and completeness. Using the MTurk platform to administer surveys significantly reduces cost, response time, inaccuracy, and barriers to access for specific patient populations, providing a more holistic snapshot of the overall US population.

Responses were crowdsourced for a 5-minute open survey that collected data on several themes: opinions on travel, CDC guidelines and adherence, ethical concerns, resource use, proactive actions, social distancing, and public perception of several facets of the COVID-19 pandemic in the United States (Multimedia Appendix 1). Regarding the public perception of severity, concern, and financial impact, study participants were asked to provide a utility score on a 10 cm vertical visual analog scale (VAS) between 0 cm-10 cm, with the scale described in detail for each question to maximize clarity of the scale being used. All collected utility scores were analyzed across age, sex, household income, and individual health risk factors such as the presence of chronic comorbidities. Questionnaire items were not randomized, and adaptive questions were used to reduce the number and complexity of survey questions. There were 2-12 questionnaire items per page, and the survey was distributed over 11 pages. Participants were able to review and change their answers. Only completed surveys were analyzed, and completeness was assessed after all surveys were collected.

Institutional review board approval was not sought or necessary for this study, as in the case of MTurk, no identifiable private or personal information was recorded and no direct interaction or engagement with any of the study participants occurred. The methodological soundness of the manuscript was assessed using the CHERRIES (Checklist for Reporting Results of Internet E-Surveys; Multimedia Appendix 2) [15].

### Screening Questions

Although Amazon MTurk requires that registered volunteers be older than 18 years, individuals may not be providing accurate demographic information when creating their account. To ensure that all surveyed participants were considered adults, the first questions of the survey asked the participants to confirm their age. No other screening questions were administered to maintain a truly diverse representation of the US population.

### Attention Check Question

To ensure that survey participants were paying close attention to each question and scenario, and that the generated data was a valid representation of patient opinions, the following attention check mechanism was included at two points in the survey [16]. Study participants were asked in two different sections (on separate pages, where participants could not go backwards to change their answer) whether they were currently taking Plaquenil. Any discordance between the two questions resulted in exclusion from this study. Those that were excluded were prevented from ever taking this survey again.

### Statistics

Data from the survey was collected and managed using REDCap (Research Electronic Data Capture; Vanderbilt University) and pooled and analyzed using Microsoft Excel 2016 (Microsoft Corporation). Statistics were performed using R Statistical Package (R Foundation for Statistical Computing) with continuous data evaluated using two-tailed two-sample unequal variances *t* tests (α=.05) assuming a normal distribution for comparisons between two groups and with analysis of variance with post hoc analysis with Tukey test, which allows for pairwise comparisons for comparisons between more than two groups. Categorical data was evaluated using chi-square testing. All responses were also screened by the Amazon MTurk platform and manually by the authors using the REDCap platform for completeness, validity, and duplicate responses.

## Results

### Study Participants

A total of 1107 MTurk participants were surveyed. Of these, 64 (5.8%) were excluded because they did not complete the survey, and 40 (3.6%) were excluded because they failed the attention check question. An additional 17 individuals were screened to have entered duplicate responses (identified by worker ID on the MTurk platform) and were deleted from the REDCap database (34 total responses). A total of 969 participants met inclusion criteria, were screened for validity and completeness, did not fail the attention check question, and were included in this study.

**Figure 1:**
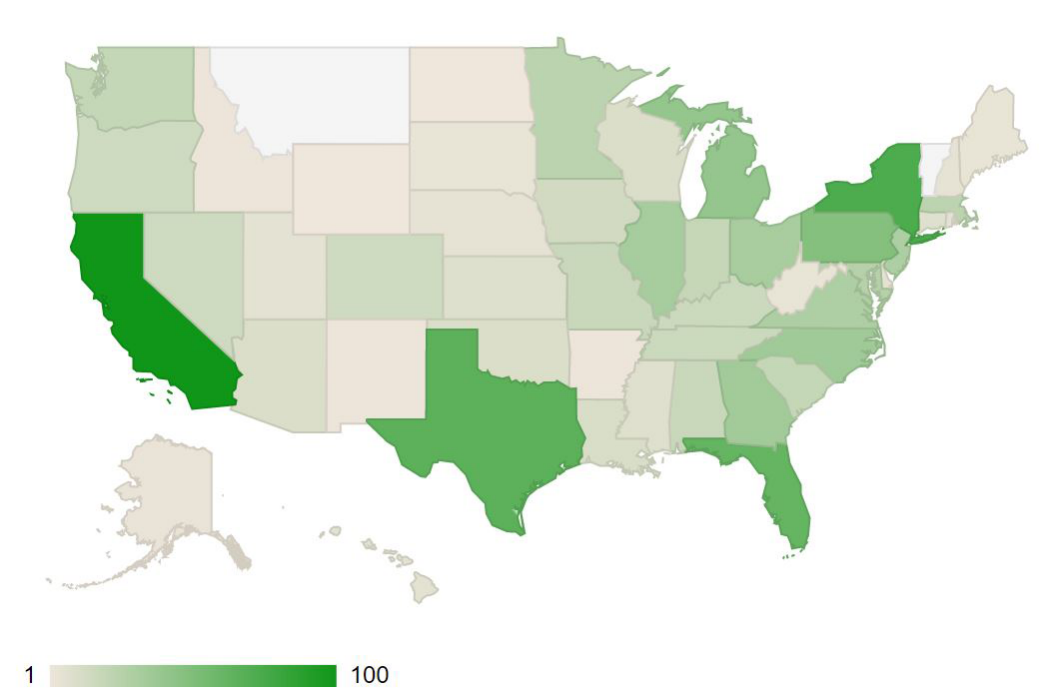
Distribution of survey respondents across the United States, with color representation of total survey impact: dark green represents the largest number of survey participants (California, n=100) and light beige the fewest with only a single respondent (Wyoming and North Dakota). There were no respondents from Montana or Vermont (white).

The MTurk human intelligence platform aims to capture a diverse number of study participants across age, sex, ethnicity, and income. Our study participants included 410 males and 559 females. The majority of the 969 participants were between the ages of 18-39 years (n=538, 55.5%), with the remaining between the ages of 40-64 years (n=366, 37.8%) or older than 65 years (n=65, 6.7%). The survey also captured a diverse representation of the United States—participants were from every state except for Montana and Vermont, with the majority from California, Florida, Texas, and New York (Figure 1).

Ethnicity was derived using the guidelines set forth by the US census bureau. Of the 969 participants, a total of 11 (1.1%) participants identified as American Indian or Alaskan Native, 91 (9.4%) as Asian, 108 (11.1%) as Black or African American, and 753 as White or Hispanic (77.6%). With respect to ethnicity, our subject population is extremely representative of the US population, as the 2019 US census demonstrated 76.3% of the population as White or Hispanic, 13.4% as Black or African American, 5.9% as Asian, and 1.3% as American Indian or Alaska Native (Multimedia Appendix 3) [17].

In addition to both accurate geographic and cultural representation with respect to the US population, the study demonstrated a median household income of US $52,000 across all study participants, which is similar to the 2019 census bureau figure of US $60,293 [17]. The distribution of income across all study participants can be viewed in Multimedia Appendix 4.

**Figure 2:**
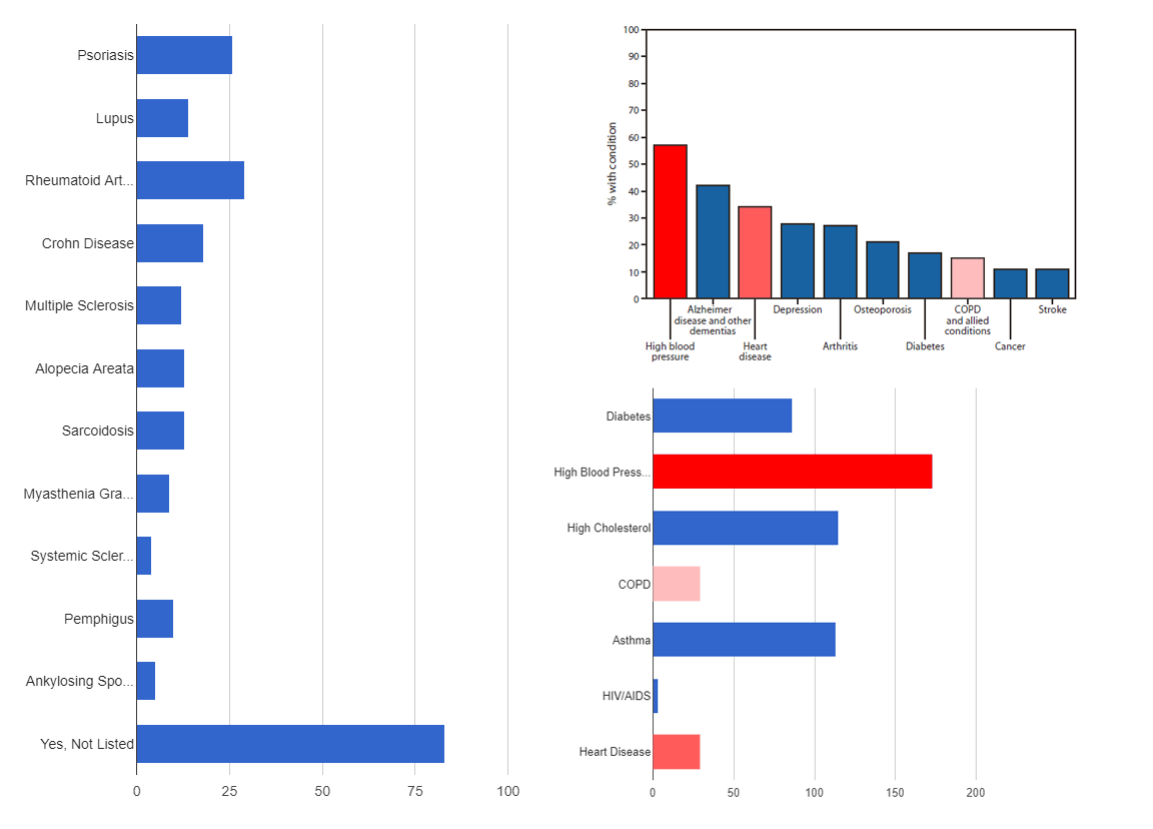
Total number of survey participants that indicated they have either a chronic health condition (n=390) or an autoimmune disorder that may increase susceptibility and risk to COVID-19 infection (n=169), and subsequent morbidity and mortality, in comparison to the ten most common chronic health conditions among persons living in residential care facilities (as of the national survey of residential care facilities in the United States in 2010), published by the Centers for Disease Control and Prevention on August 10, 2012, as reported by Caffrey et al (upper right). Darker red corresponds to the most common and lighter red to the least common chronic health conditions. COPD: chronic obstructive pulmonary disease.

Study participants were asked to disclose pertinent medical history that may put them at higher risk of morbidity and mortality from COVID-19. Out of 969 participants, a total of 367 (37.9%) indicated they had a chronic medical condition including diabetes, high blood pressure, high cholesterol, chronic obstructive pulmonary disease (COPD), asthma, HIV/AIDS, or chronic heart disease. An additional 145 (15.0%) participants indicated a history of one or more autoimmune conditions including psoriasis, lupus, rheumatoid arthritis, Crohn disease, multiple sclerosis, alopecia areata, sarcoidosis, myasthenia gravis, systemic sclerosis, pemphigus, ankylosing spondylitis, or other conditions not listed (Figure 2). Participants did not divulge whether the disease was currently active. However, 49 (5.1%) indicated they were currently taking oral steroid medication, 22 (2.27%) participants indicated they were a recipient of a recent organ transplantation requiring current immunosuppressive medication, and 18 (1.86%) subjects indicated they were currently being treated with chemotherapy. There were 187 (19.3%) participants self-identified as currently smoking tobacco. In total, 508 (52.4%) high-risk individuals were identified.

### Mental Health Impact

**Figure 3:**
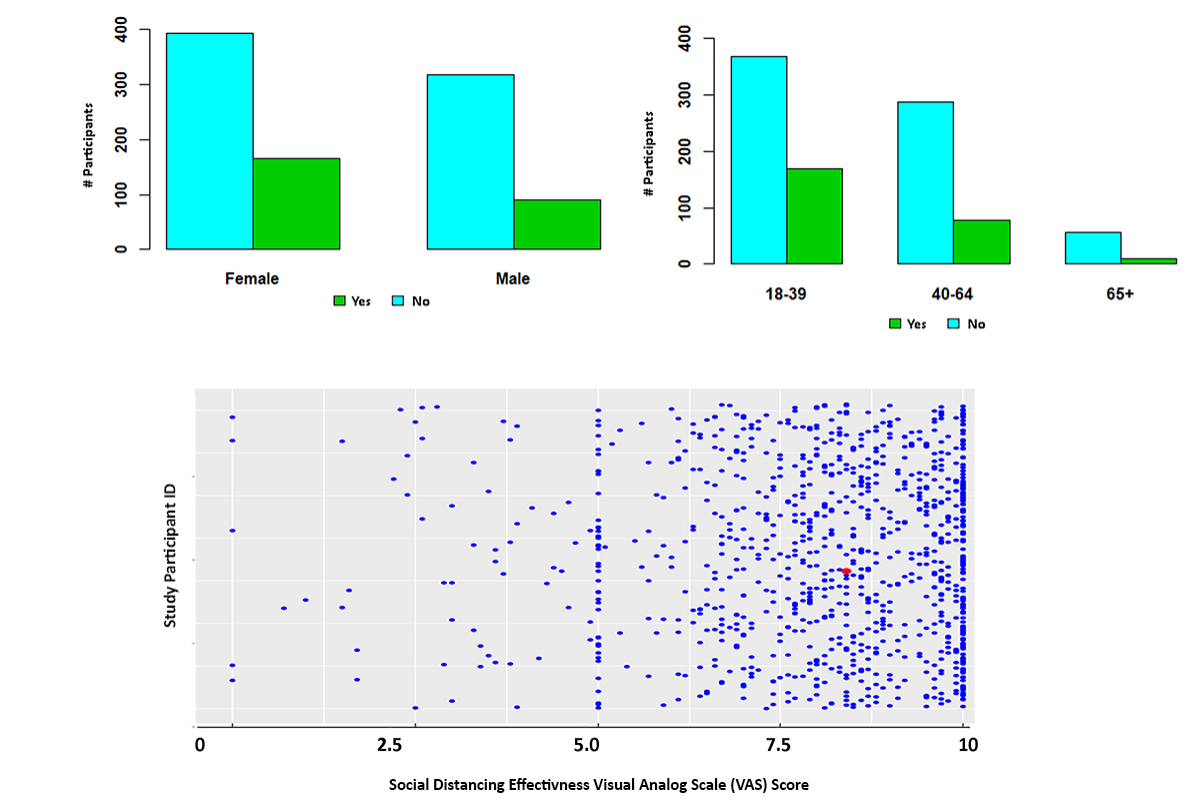
Survey response to “has social distancing had a negative impact on your mental health” question across sex and age cohorts, and a scatter plot depicting the visual analog scale scores of how effective people believe social distancing is at addressing the spread of COVID-19 (where 0 is not effective at all, and 10 is most effective), with a mean of 7.98 (SD 1.94) and a median of 8.40 (red dot).

Study participants were asked if social distancing has had a negative impact on their mental health (Figure 3). Pearson chi-square test demonstrated a significant mental health impact in those aged 18-39 years when compared to those ≥40 years (*P*<.001). Pearson chi-square test with Yates continuity correction also demonstrated a significantly higher negative impact on the mental health of female participants as compared to males (*P*=.01).

### Severity Perception

**Figure 4:**
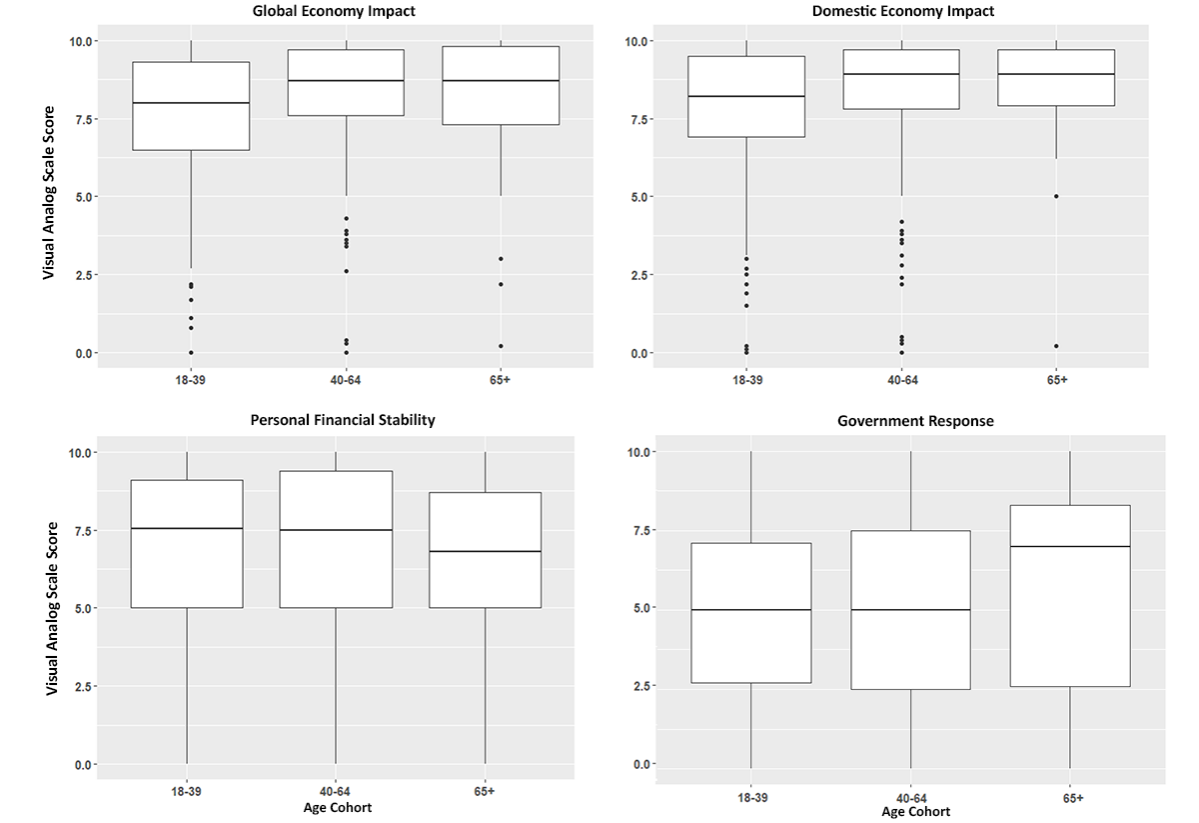
Box and whisker plots depicting the distribution and median of visual analog scale scores across all study participants with respect to age cohorts in response to how concerned individuals are about the negative impact of COVID-19 on the overall global economy (with 0 representing no concern at all and 10 the most concern), how concerned individuals are about the negative impact of COVID-19 on the overall US economy, how concerned individuals are about the negative impact of COVID-19 on personal financial stability, and how appropriately do individuals believe the US government has responded to the COVID-19 pandemic (where 0 indicates that they do not think the government or Centers for Disease Control and Prevention [CDC] has responded appropriately at all and 10 indicates that they think the government and CDC have responded perfectly).

Study participants were asked to indicate on a VAS scale (from 0 to 10, where 10 represented the highest level of severity) how severe they believe the COVID-19 outbreak to be, considering published data from the CDC and their own perceptions of the severity (Figure 4). Participants were provided an infographic that demonstrated the number of cases across the United States and information about the case mortality rate.

It was found that age had a linear correlation to perceived severity (*F*_2_=8.21, *P*<.001). Those aged 18-39 years indicated a mean VAS of 7.05 (95% CI 6.88-7.22, the lowest severity), those aged ≥65 years reported a mean VAS of 8.06 (95% CI 7.59-8.54, the highest severity), and those aged 40-64 years demonstrated a mean VAS of 7.40 (95% CI 7.17-7.63). The difference in perceived severity was statically significant when comparing those younger than 40 years to those 65 years or older (*P*<.001) and when comparing those younger than 40 years to those aged 40-64 years (*P*=.04). The difference in perceived severity did not differ between those aged 40-65 years and those aged ≥65 years (*P*=.05). Reported worry about personal health (indicated by rating on VAS scale from 0 to 10, where 0 represented no worry and 10 represented extreme worry) did not significantly differ across age groups (*P*=.13). Those aged 18-39 years indicated a mean VAS of 5.73 (95% CI 5.50-5.96), those aged 40-64 years reported a mean VAS of 6.07 (95% CI 5.78-6.36), and those aged ≥65 years demonstrated a mean VAS of 6.17 (95% CI 5.45-6.90).

It was found that the perceived severity of the COVID-19 pandemic significantly differed between men and women (t_834.22_=3.942, *P*<.001). Men indicated a mean VAS of 6.94 (95% CI 6.72-7.15), and women indicated a mean VAS of 7.48 (95% CI 7.31-7.65).

The perceived severity of the COVID-19 pandemic did not differ between high-risk and low-risk participants (t_966.91_=–1.1766, *P*=.24), where high risk was defined as a participant with any of the following: at least one chronic medical condition including diabetes, high blood pressure, high cholesterol, COPD, asthma, HIV/AIDS, or chronic heart disease; a history of any autoimmune condition including psoriasis, lupus, rheumatoid arthritis, Crohn disease, multiple sclerosis, alopecia areata, sarcoidosis, myasthenia gravis, systemic sclerosis, pemphigus, ankylosing spondylitis, or other conditions not listed; current oral steroid medication use; recipient of a recent organ transplantation requiring current immunosuppressive medication; current treatment with chemotherapy; or current tobacco smoker. High-risk participants indicated a mean VAS of 7.33 (95% CI 7.13-7.52) and low-risk participants reported a mean VAS of 7.17 (95% CI 6.98-7.35) with respect to perceived severity. However, high-risk individuals were more likely to believe that they would become infected with COVID-19 (*P*=.005) and experience serious illness or die due to infection with COVID-19 (*P*<.001). Furthermore, 32.7% (166/508) of high-risk individuals and 24.3% (112/461) of low-risk individuals believed they would become infected with COVID-19, and 15.6% (79/508) of high-risk individuals and 7.81% (36/461) of low-risk individuals believed they would become seriously ill or die due to COVID-19.

When stratifying participants by annual income (US $0-US $25,000; US $25,001-US $50,000; US $50,001-US $75,000; US $75,001-US $100,000; and greater than US $100,000), no significant difference in perceived severity was observed across any income cohort (*F*_4_=0.33, *P*=.86).

### Government Response Perception

Study participants were asked to provide their opinion regarding the appropriateness of the US government’s response to the novel coronavirus pandemic (as of March 24, 2020) by way of a VAS scale from 0 to 10, where 0 represented an inappropriate response and 10 represented an appropriate response. It was found that the perceived appropriateness of the US government’s response significantly differed across age groups (*F*_2_=3.663, *P*=.03). Those aged 18-39 years indicated a mean VAS of 4.85 (95% CI 4.62-5.08, the lowest appropriateness), those age ≥65 years reported a mean VAS of 5.78 (95% CI 4.96-6.60, the highest appropriateness), and those aged 40-64 years demonstrated a mean VAS of 5.16 (95% CI 4.86-5.46). The difference in perceived appropriateness of government response was statically significant when comparing those younger than 40 years to those aged ≥65 years (*P*=.04) but not when comparing those younger than 40 years to those aged 40-64 years (*P*=.25) or those aged 40-64 years to those aged ≥65 years (*P*=.24).

### Economic Impact

**Figure 5:**
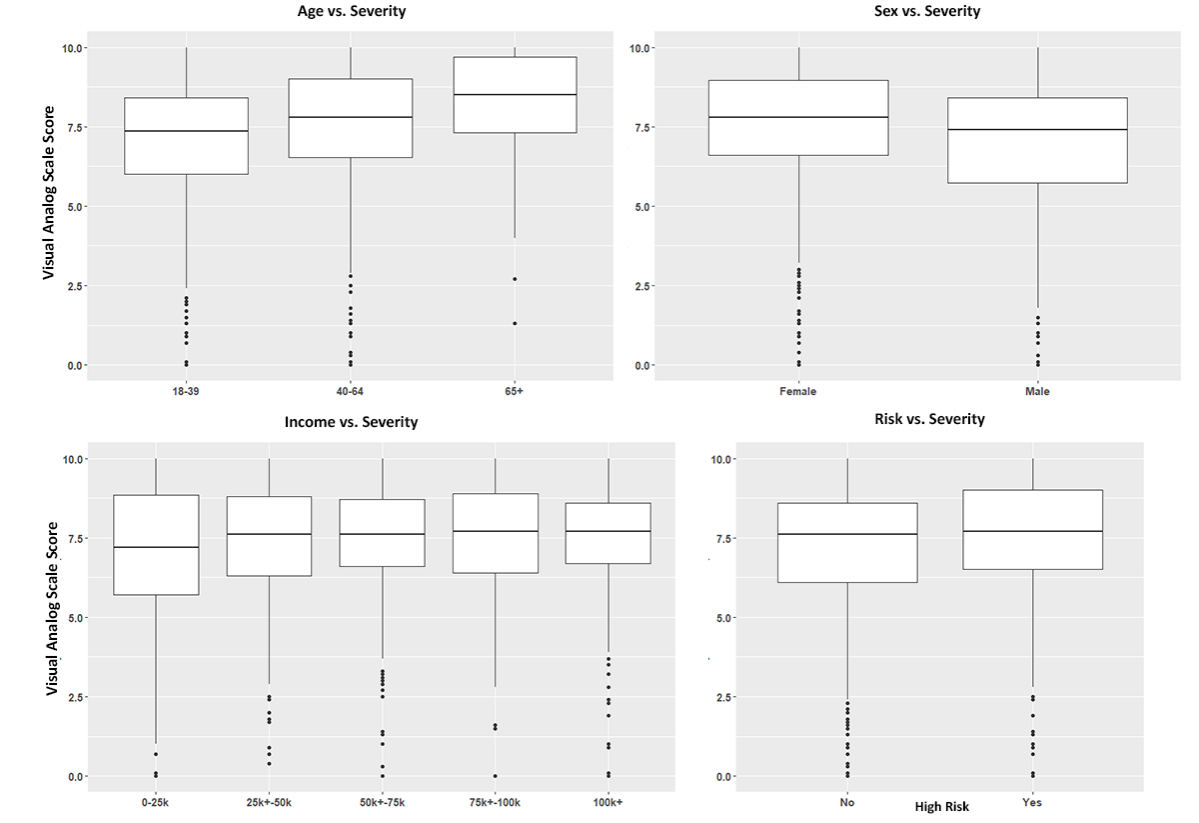
Box and whisker plots depicting the distribution and median of visual analog scale scores of study participants in response to their perception of the severity of the COVID-19 pandemic, with respect to age cohort, sex, income level, and health risk status, with high-risk individuals defined within the text.

Study participants were asked to indicate on a VAS scale (from 0 to 10, where 0 represented no concern and 10 represented the highest level of concern) their concern regarding the effect of the COVID-19 outbreak on their personal financial stability, the US economy, and the global economy (Figure 5).

The reported concern regarding personal financial stability was not found to differ significantly between age groups (*F*_2_=0.743, *P*=.48). Those aged 18-39 years reported a mean VAS of 6.96 (95% CI 6.74-7.18), those aged 40-64 years indicated a mean VAS of 6.92 (95% CI 6.64-7.20), and those aged ≥65 years demonstrated a mean VAS of 6.53 (95% CI 5.85-7.21).

The reported concern with respect to the effect of COVID-19 on the US economy was found to significantly differ between age groups (*F*_2_=11.74, *P*<.001). Those aged 18-39 years indicated a mean VAS of 7.86 (95% CI 7.69-8.03, lowest concern), those aged ≥65 years indicated a mean VAS of 8.52 (95% CI 8.12-8.93, highest concern), and those aged 40-64 years indicated a mean VAS of 8.43 (95% CI 8.25-8.61). The difference in perceived concern regarding the US economy was statically significant when comparing those younger than 40 years to those aged 40-64 years (*P*<.001) and when comparing those younger than 40 years to those aged ≥65 years (*P*=.02). When comparing those aged 40-64 years to those aged ≥65 years, no significant difference was observed (*P*=.93).

The reported concern regarding the effect of COVID-19 on the global economy was found to significantly differ between age groups (*F*_2_=14.61, *P*<.001). Those aged 18-39 years indicated a mean VAS of 7.64 (95% CI 7.47-7.81, lowest concern), those aged 40-64 years reported a mean VAS of 8.33 (95% CI 8.14-8.51, highest concern), and those aged ≥65 years demonstrated a mean VAS of 8.23 (95% CI 7.75-8.71). The difference in perceived concern regarding the global economy was statically significant when comparing those younger than 40 years to those aged 40-64 years (*P*<.001). When comparing those younger than 40 years to those aged ≥65 years, or when comparing those aged 40-64 years to those aged ≥65 years, no significant difference was observed (*P*=.05 and *P*=.92, respectively).

### Social Distancing

Social distancing from family, friends, and coworkers was not observed to be significantly associated with age groups (*P*=.15). Of the 538 participants aged 18-39 years, 488 (90.7%) indicated that they are minimizing contact with family, friends, and coworkers, and 50 (9.3%) indicated that they are not. Of the 366 participants aged 40-64 years, 340 (92.9%) reported that they are minimizing contact with family, friends, and coworkers, and 26 (7.1%) reported that they are not. Of the 65 participants aged ≥65 years, 63 (96.9%) indicated that they are minimizing contact with family, friends, and coworkers, and 2 (3.1%) indicated that they are not.

Social distancing from family, friends, and coworkers was observed to be significantly associated with sex (*P*=.001). Of the 559 female participants, 528 (94.4%) indicated that they are minimizing contact with family, friends, and coworkers, and 31 (5.6%) indicated that they are not. Of the 410 male participants, 363 (88.5%) reported that they are minimizing contact with family, friends, and coworkers, and 47 (11.5%) reported that they are not.

### CDC Guidelines Adherence

It was found that 21 (2.2%) of the 969 study participants were currently diagnosed with COVID-19 by a medical health professional. It was also found that 13 (61.9%) of these patients were prescribed and taking Plaquenil as a treatment. This is not a CDC guideline, and this was not associated with any such guidelines within the survey instrument. It has only been included here for reference, as several studies have shown potential efficacy of treatment with hydroxychloroquine, and this information has been widely distributed by mainstream media [18,19].

Participants were asked to provide answers to a series of questions to determine if sanitary and social distancing guidelines were being followed as recommended by the CDC (as of March 24, 2020) [1]. It was found that, of the 969 participants, 92.3% (n=894) of individuals would stay home if they were sick unless they needed to seek medical care; 91.2% (n=884) would wear a face mask when around other people and before entering a health care provider’s office; 85.8% (n=831) avoid touching their eyes, nose, and mouth with unwashed hands; 95.4% (n=924) indicated that they are avoiding close contact with people who are sick; 95.5% (n=925) throw used tissues in the trash; 92.6% (n=897) indicated they would wash hands with soap and water often for at least 20 seconds; and 87.8% (n=851) would use hand sanitizer that contains at least 60% alcohol if soap and water were not readily available. Over three-fourths (n=769, 79.4%) of study participants indicated that they wash their hands immediately after coughing or sneezing, and 73.7% (n=714) indicated that they clean and disinfect frequently touched surfaces daily. Additionally, 82.1% (n=796) clean surfaces that are visibly dirty with detergent or soap prior to disinfection.

A majority (n=615, 63.5%) of the 969 study participants indicated that they would not wear gloves or a face mask when going to public places if they were not sick. However, 50.2% (n=486) of study participants believe that the use of gloves or face masks by the general population helps prevent the spread of COVID-19.

**Figure 6:**
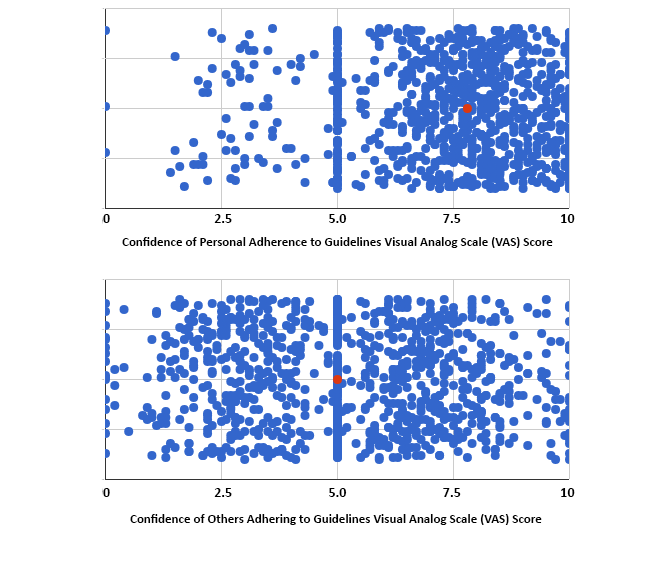
Scatter plot depicting visual analog scores of all study participants, demonstrating the perception of confidence in personal ability to minimize the risk of infection, with a mean of 7.51 (SD 1.93) and a median of 7.80 (red dot), and confidence that others are adhering to the Centers for Disease Control and Prevention guidelines to minimize the likelihood of COVID-19 infection spread, with 0 representing no confidence and 10 the most confidence, with a mean of 5.28 (SD 2.35) and a median of 5.0 (red dot).

Participants were asked to indicate their confidence, where 0 represented no confidence and 10 represented full confidence, regarding their personal ability to minimize becoming infected with COVID-19. The participants demonstrated a mean of 7.53 (95% CI 7.41-7.65). Regarding their confidence in other people following guidelines to prevent the likelihood of COVID-19 spread, participants reported a mean of 5.26 (95% CI 5.11-5.40), which was significantly lower when compared to participant confidence in their personal ability (t_1864.4_=–23.572, *P*<.001). The majority of the 969 participants (n=717, 74%) believed that those younger than 40 years are contributing to the spread of COVID-19 more so than those 40 years or older (Figure 6).

### Ethical Considerations

Study participants were asked several yes or no questions about ethical considerations with respect to resource management and the availability of medical care. When asked if they were buying or would buy excess amounts of sanitation supplies (ie, hand sanitizer, sanitizing wipes, and hand soaps) despite knowing there may not be enough supplies for everyone, 89 (9.2%) of the 969 indicated that they were or would, and 90.8% (n=880) reported that they were not or would not. When buying essential supplies, study participants (n=715, 73.8%) did not believe that people take the needs of others into consideration (Multimedia Appendix 5).

When asked if they would pay for medications they might need, even if they were not currently sick, 199 (20.5%) of the 969 participants indicated they would do so. Similarly, 171 (17.6%) reported that even if they were not sick, they would buy and use face masks with active knowledge of the limited supply available to health care workers and sick people. Although the majority (n=775, 80.0%) indicated they were not upset when asked if they were upset that they could not see their physicians in person for reasons other than COVID-19–related concerns, knowing that in-person exposure would increase their risk of acquiring the virus, 194 (20.0%) reported they were upset they could not see their physician.

### Travel Considerations

Study participants were asked additional yes or no questions about their thoughts regarding travel. The majority of the 969 responders indicated that they both have not traveled outside of the United States within the previous 3 months (n=901, 93.0%) and did not have plans to travel outside of the United States within the coming 3 months (n=917, 94.6%). Of the 52 (5.4%) participants who reported plans to travel internationally, 28 (53.8%) planned on cancelling their arrangements, though 24 (46.2%) did not. The majority of participants (n=558, 58.6%) believed that US citizens should be allowed to return to the United States, but 401 (41.4%) did not. The majority (n=651, 67.2%) indicated that non-US citizens currently in the United States should be allowed to return to their countries of origin.

The majority of the 969 participants (n=734, 75.9%) indicated that they did not plan on travelling domestically within the United States in the next 3 months. Of the 234 (24.1%) participants who reported planning on travelling domestically, 136 (58.1%) did not plan on cancelling their arrangements. The majority of participants (n=550, 56.8%) indicated that individuals and families should not be allowed to travel domestically within the United States. A total of 794 (81.9%) believed that those younger than 40 years should cancel travel plans, and 859 (88.6%) indicated that those 40 years or older should cancel travel plans. When asked how important travel is as it relates to the current COVID-19 pandemic, where 0 was of no importance at all and 10 was of the most importance, the mean response was 6.87 (95% CI 6.68-7.06).

### Proactive Measures

Study participants were asked yes or no questions about several proactive measures they may or may not have taken regarding avoiding infection and stocking supplies. When asked if they have taken proactive measures to protect their health, the majority of the 969 participants responded “yes” (n=921, 95.0%), and 17 (1.75%) indicated that they have started taking Plaquenil as a preventative measure.

Of the 701 (72.3%) employed participants, 440 (62.8%) indicated that they were working from home. Of the 261 employed participants not working from home (37.2% of employed participants), the majority (n=199, 76.2%) stated they were not working from home because their employers had not made that option available (Multimedia Appendix 6).

The majority of the 969 respondents (n=737, 76.1%) indicated that they had stocked up on essential supplies such as food, sanitizing products, and necessary medications for chronic health conditions. The majority of respondents (n=693, 71.5%) believed that they had enough essential supplies for all individuals in their household, and 869 (89.7%) indicated that other people were proactively buying too many essential supplies. A total of 473 (48.8%) respondents believed that there are enough essential supplies for all individuals and families in the United States while 496 (51.2%) did not. The majority of respondents (n=715, 73.8%) did not believe individuals take the needs of others into account when purchasing essential supplies.

When asked in how many of the last 10 days had they been concerned that they would be unable to obtain resources such as food, sanitizing products, and necessary medications for themselves or their families, 230 (23.7%) of 969 participants responded that they had not been concerned, and 153 (15.8%) responded that they had been concerned every day in the past 10 days.

## Discussion

### Principal Results

The study showed several key differences in perception. Older individuals are at a higher risk of morbidity and mortality from COVID-19 infection, and their perception of the crisis was demonstrably more severe than that of the younger population (aged <40 years). Males were less concerned than females regarding the severity of the pandemic and were less likely to practice social distancing. Although there is no established difference in sex with regard to susceptibility to COVID-19 infection, there is limited epidemiological data to suggest increased burden of morbidity and mortality in males [20]. The role of differences in perceived severity of the pandemic and social distancing practices between males and females may contribute to this increased susceptibility because males may underestimate the threat of COVID-19 and engage less rigorously in social distancing.

The mental health implications of social distancing were greatest for younger people despite being at lower risk of morbidity and mortality as compared to older people. As social distancing has resulted in many social interactions becoming confined to the internet, it would be expected that younger people would be least affected by this transition, as younger generations are commonly more adept at using technology and traditionally more associated with the use of social media as a component of their social interactions. However, it may be possible that, because younger people experience a greater proportion of online interactions, they may place a larger relative value on in-person interactions than do older individuals. Because social distancing has resulted in a quick and dramatic reduction of in-person interactions, this may have disproportionately affected younger people. It is also possible that younger people are less likely to live with others when compared to those 40 years or older. This would mean that, during this period of social distancing when people are largely confined to their homes, young people are disproportionately isolated compared to older people. However, information regarding cohabitants was not collected in this survey. The larger negative mental health impact due to social distancing observed in younger people may also be due to their arguably greater potential loss of income. Interestingly enough, there was no significant difference observed between income groups, so financial factors alone cannot explain the fear and emotional toll that younger individuals are reportedly experiencing.

Though it was expected that high-risk individuals would perceive this pandemic as more severe than low-risk individuals, no significant difference was observed. Higher severity VAS scores were anticipated among high-risk participants because, by definition, they are at elevated risk for morbidity and mortality from infection. It is possible that a portion of high-risk individuals are not aware of their augmented risk or that a portion of low-risk individuals mistakenly believe they are at elevated risk. The collected data indicates that the public may be misevaluating the threat of morbidity and mortality of COVID-19. It is conceivable that this misalignment between perceived and actual threat may lead to noncompliance with guidelines, especially if one believes their risk to be less than it actually is. This may also lead to a disproportionate hoarding of resources among those at lower risk relative to those at higher risk and may have needless deleterious effect on mental health, especially if one believes their risk to be greater than it actually is.

The data collected here suggests that younger people believe the US government has responded to this crisis inappropriately while older people appear to be more supportive of the government’s actions. However, the mean VAS response for all age groups was less than 6.0 (scale from 0-10), suggesting that approval of government response is low regardless of age. Although all age groups indicated concern regarding their personal finances, this concern did not differ between age groups. However, older individuals appear to be more concerned about the United States and global economy than are younger people.

Though many may think they know what practices to adopt to prevent viral spread, many participants said that if they were infected they would not wear a mask in public. This indicates that the public may be largely unaware of their lack in knowledge regarding the transmissibility of COVID-19 or that they are indifferent to contributing to the spread of COVID-19. Additionally, although many participants assumed that they knew how to minimize viral spread, they also assumed that others did not, and the degree to which they were confident in their own ability to reduce viral spread was significantly greater than the degree to which they believed other people could contribute to minimizing viral transmission. This may make disseminating accurate information and changing deleterious practices more difficult, as the data here suggests people are likely to think they are engaging in safe behavior when they might not be and that they believe their behavior is appropriate, while the actions of others are not.

Although hoarding practices are widely known to be disruptive to care, many admitted they would purchase unneeded materials and medicines despite the deleterious impact on public health. Furthermore, though the majority stated they have enough essential supplies for themselves and family, less than half believed there were enough essential resources for everyone, and the majority believed that people do not account for the needs of others when securing essential resources. This data suggests that possibly underlying hoarding behavior is a fear of the finiteness of resources and a lack of trust in others to responsibly ration.

Interestingly, though the spread of COVID-19 across boarders has been in part attributed to travel, of those with travel plans both foreign and domestic, 40%-60% planned to continue travelling. This may be indicative of a fatigue created by social distancing that, due to a newly imposed relative isolation, people are wanting to travel despite the risks of contracting and spreading viral infection. This may also reflect the need to travel to self-isolate with family and loved ones, rather than to do so alone. There may also be a nonnegligible portion of the population that remains unaware of the effect of travelling on viral spread.

### Limitations

Although crowdsourcing is an extremely powerful tool, limitations exist. The potential limitation of using Amazon MTurk may be that study participants could submit multiple survey responses. Individuals could also circumvent the survey process completely by using a random number generator to create survey completion codes that are required for study participants to claim their wages. However, this did not have any impact on the overall study results, as all surveys were screened for completeness, quality, uniqueness, and accuracy, and duplicated responses were excluded. In addition, all completion codes were screened for authenticity, and time from survey start to survey completion was measured.

Inherent to many surveying methodologies, there also exists an internal bias with respect to those individuals who choose to take a survey about COVID-19. Those who have been impacted more severely or closely by the global implications of this pandemic may be more inclined to disseminate their opinions. The financial interest of the study participants may pose another potential limitation, yet the authors believe that this does not significantly skew the study results, with participants being compensated a total of US $0.25 for their response. In addition, the questions within the survey are not easily influenced by personal financial gain. However, MTurk remains a powerful platform in its ability to crowdsource an accurate representation of the US population as demonstrated by our demographic data being extremely similar to the data published in 2019 by the US Census Bureau. This has also been reflected by many prior crowdsourcing studies using the MTurk tool [21-25].

### Conclusions

The authors believe that COVID-19 is contributing to tremendous emotional, financial, and health-related stress. This study helps to elucidate some of the public’s fears about this disease as well as several key areas where better education may help improve outcomes. Namely, there needs to be a targeted effort to inform those that are infected about the importance of isolation and personal protective equipment. Furthermore, efforts should target hoarding practices and travel. Age-related differences in the perception of severity may change with time, and there is no good intervention to prevent these at this time. The emotional toll will be great, and the negative impact on mental health may continue to increase, especially in the younger population, though the long-term effects of this pandemic will be difficult to predict. Campaigns to target those in need of mental health resources now may curb the long-term negative mental health effects of this pandemic. In the future, this study will be repeated to evaluate how perception has changed over time and to better determine the long-term effects on the general population. Several efforts have already been made to continue to understand public opinions across race and ethnicity [26].

## Appendix

Multimedia Appendix 1 Survey instrument used for data collection.

Multimedia Appendix 2 CHERRIES (Checklist for Reporting Results of Internet E-Surveys).

Multimedia Appendix 3 Comparison of the distribution of ethnicity across all study participants (n=969) and the 2019 US census data published by the US Census Bureau.

Multimedia Appendix 4 Scatter plot depicting the annual household income of each individual study participant included in our analysis (n=969).

Multimedia Appendix 5 Answers to yes or no questions asked to all study participants (n=969) with respect to ethical considerations of stockpiling or hoarding materials and other societal considerations.

Multimedia Appendix 6 Answers to yes or no questions asked to all study participants (n=969) with respect to proactive measures taken in response to the COVID-19 pandemic.

## Abbreviations

CDC: Centers for Disease Control and Prevention
CHERRIES: Checklist for Reporting Results of Internet E-Surveys
COPD: chronic obstructive pulmonary disease
MTurk: Mechanical Turk
REDCap: Research Electronic Data Capture
VAS: visual analog scale
