# Supplementary material for: The Public’s Perception of the Severity and Global Impact at the Start of the SARS-CoV-2 Pandemic: A Crowdsourcing-Based Cross-Sectional Analysis
Source: J Med Internet Res. 2020 Nov 26;22(11):e19768. doi: 10.2196/19768 (PMC7695545; doi:10.2196/19768)
Supplement: Multimedia Appendix 1 [file jmir_v22i11e19768_app1.pdf]

COVID-19 Public Survey

Please complete the survey below about the current coronavirus pandemic.

Approximate Length: 5 Minutes

Thank you!

Which age group are you in?	◯ 18-39 ◯ 40-64 ◯ 65+
What is your sex?	◯ Male ◯ Female

What state are you currently located?	◯ Alabama ◯ Alaska<br>◯ Arizona ◯ Arkansas<br>◯ California ◯ Colorado<br>◯ Connecticut ◯ Delaware<br>◯ Florida ◯ Georgia<br>◯ Hawaii ◯ Idaho ◯ Illinois<br>◯ Indiana ◯ Iowa ◯ Kansas<br>◯ Kentucky ◯ Louisiana<br>◯ Maine ◯ Maryland<br>◯ Massachusetts ◯ Michigan<br>◯ Minnesota ◯ Mississippi<br>◯ Missouri ◯ Montana<br>◯ Nebraska ◯ Nevada<br>◯ New Hampshire ◯ New Jersey<br>◯ New Mexico ◯ New York<br>◯ North Carolina ◯ North Dakota<br>◯ Ohio ◯ Oklahoma<br>◯ Oregon ◯ Pennsylvania<br>◯ Rhode Island ◯ South Carolina<br>◯ South Dakota ◯ Tennessee<br>◯ Texas ◯ Utah ◯ Vermont<br>◯ Virginia ◯ Washington<br>◯ West Virginia ◯ Wisconsin<br>◯ Wyoming
What is your ethnicity?	◯ American Indian or Alaska Native<br>◯ Asian<br>◯ Black or African American<br>◯ Native Hawaiian or Other Pacific Islander<br>◯ White
What is your approximate total yearly household income?	________________________
Do you currently take oral steroid medications?	◯ Yes ◯ No
Have you been diagnosed with any of the following chronic medical conditions (check all that apply)?	☐ Diabetes<br>☐ High Blood Pressure<br>☐ High Cholesterol<br>☐ COPD<br>☐ Asthma<br>☐ HIV/AIDS<br>☐ Heart Disease
Have you been diagnosed with any of the following autoimmune conditions, requiring current treatment with immunosuppressive drugs (check all that apply)?	☐ Psoriasis<br>☐ Lupus<br>☐ Rheumatoid Arthritis<br>☐ Crohn's Disease<br>☐ Multiple Sclerosis<br>☐ Alopecia Areata<br>☐ Sarcoidosis<br>☐ Myasthenia Gravis<br>☐ Systemic Sclerosis<br>☐ Pemphigus<br>☐ Ankylosing Spondylitis<br>☐ Yes, Not Listed
Have you recently been a recipient of an organ transplant, requiring current treatment with immunosuppressive medication?	◯ Yes ◯ No

Are you currently being treated with chemotherapy for cancer?	◯ Yes ◯ No
Do you smoke tobacco?	◯ Yes ◯ No
Are you currently taking plaquenil (treatment for COVID-19)?	◯ Yes ◯ No

Page 1/6

On a scale from 0-10, where 0 is no importance at all, and 10 is the most importance, what is your perception of the importance of travel as it relates to this disease?

Please move the slider up or down to best reflect your opinion.

0 (Least Important) 10 (Most Important)

(Place a mark on the scale above)

Have you traveled outside of the US in the last three months?	◯ Yes ◯ No
Do you plan on traveling outside the US in the next 3 months?	◯ Yes ◯ No
Do you plan on canceling your foreign travel plans?	◯ Yes ◯ No
Do you plan on traveling domestically in the next 3 months?	◯ Yes ◯ No
Do you plan on canceling your domestic travel plans?	◯ Yes ◯ No
Do you think that young people (age less than 40 years-old) should cancel travel plans?	◯ Yes ◯ No
Do you think that older people (age 40 years-old and above) should cancel travel plans?	◯ Yes ◯ No
Do you think that US citizens outside of the country should be allowed to return at the current moment?	◯ Yes ◯ No
Do you think that non-US citizens should be allowed to return home to their country of origin?	◯ Yes ◯ No
Do you think that individuals or families should be able to travel within the US?	◯ Yes ◯ No

Page 2/6

Have you taken proactive measures to protect your health? Examples include, washing hands often, avoiding large crowds, limiting travel, working from home, avoiding public transport, minimizing contact with others, and covering nose or mouth when sneezing or coughing.	○ Yes ○ No
Have you stocked up on essential supplies such as food, sanitizing products, and necessary medications for chronic health conditions?	○ Yes ○ No
Are you currently employed?	○ Yes ○ No
Are you currently working from home?	○ Yes ○ No
Are you not working at home because your employer has not made that option available?	○ Yes ○ No
In how many of the last 10 days have you been concerned that you would be unable to obtain resources, such as food, sanitizing products, and necessary medications, for yourself and your family?	○ 0 ○ 1 ○ 2 ○ 3 ○ 4 ○ 5 ○ 6 ○ 7 ○ 8 ○ 9 ○ 10
Have you started to take plaquenil (treatment for COVID-19) as a preventative measure?	○ Yes ○ No
Do you believe there are enough essential supplies, such as food, sanitizing products, and necessary medications, for all individuals and families in the US?	○ Yes ○ No
Do you believe you have enough essential supplies, such as food, sanitizing products, and necessary medications, for all individuals in your household?	○ Yes ○ No
Do you believe other people are proactively buying too many essential supplies, such as food, sanitizing products, and necessary medications?	○ Yes ○ No

Page 3/6

If you are not sick would you pay for medication that you MIGHT need, even though this medication may be used for other sick people?	◯ Yes ◯ No
If you are not sick would you buy and use face masks even though there is a limited supply and they may be needed for healthcare workers and other sick people?	◯ Yes ◯ No
Are you buying excess amounts of sanitation supplies such as hand sanitizer, sanitizing wipes, hand soaps, or solutions with 60% alcohol, even though you know there may not be enough of these resources for other people?	◯ Yes ◯ No
Are you upset that you cannot see your physicians in-person for reasons other than COVID-19 related concerns?	◯ Yes ◯ No
Are you purchasing food supplies which will last longer than two weeks in anticipation of food shortages?	◯ Yes ◯ No
Do you believe other people take the needs of others into consideration when buying essential supplies, such as food, sanitizing products, and necessary medications?	◯ Yes ◯ No
If you are not sick would you pay for and use plaquenil (treatment for COVID-19), even though this medication may be used for other sick people?	◯ Yes ◯ No

Page 4/6

Are you avoiding groups of more than ten people? ◯ Yes ◯ No

Are you minimizing contact with friends, coworkers, and family? ◯ Yes ◯ No

Are you minimizing contact with friends, coworkers, and family that may be at risk? ◯ Yes ◯ No

Are you avoiding public transportation whenever possible? ◯ Yes ◯ No

Are you limiting nonessential travel? ◯ Yes ◯ No

Are you skipping nonessential social gatherings? ◯ Yes ◯ No

Are you avoiding crowded bars, restaurants, and sporting arenas? ◯ Yes ◯ No

Do you think social distancing is important for young people (less than 40 years-old) in preventing the spread of disease?
◯ Yes
◯ No

Do you think social distancing is important for older people (age 40 years-old and over) in preventing the spread of disease?
◯ Yes
◯ No

On a scale from 0-10, where 0 is not at all effective and 10 is the most effective, how effective do you think social distancing is at addressing the spread of COVID-19?

Please move the slider up or down to best reflect your opinion.

0 (Least Effective) — 10 (Most Effective)

(Place a mark on the scale above)

On a scale from 0-10, where 0 is without difficulty and 10 is the most difficult, how difficult is it for you to practice social distancing?

Please move the slider up or down to best reflect your opinion.

0 (No Difficulty) — 10 (Most Difficulty)

(Place a mark on the scale above)

Has social distancing had a negative impact on your mental health? ◯ Yes ◯ No

Have you required mental health services as a result of a negative impact on your mental health due to social distancing? ◯ Yes ◯ No

Do you think people who feel sick should stay home even if it means they cannot work or purchase essential supplies such as food, sanitizing products, and necessary medications? ◯ Yes ◯ No

Page 5/6

Are you currently sick with COVID-19 (diagnosed by a medical health professional)?	◯ Yes ◯ No
Are you currently taking plaquenil (treatment for COVID-19), as prescribed by your physician?	◯ Yes ◯ No
If you became sick, would you stay home if you are sick, except to get medical care?	◯ Yes ◯ No
If you became sick, would you go to the emergency room or see your primary care physician?	◯ Yes ◯ No
If you became sick, would you wear a facemask when you are around other people (e.g., sharing a room or vehicle) and before you enter a healthcare provider's office. If you are not able to wear a facemask (for example, because it causes trouble breathing), then would you do your best to cover your coughs and sneezes?	◯ Yes ◯ No
If you became sick, should people who are caring for you wear a facemask if they enter your room?	◯ Yes ◯ No
Do you wash your hands often with soap and water for at least 20 seconds especially after you have been in a public place, or after blowing your nose, coughing, or sneezing?	◯ Yes ◯ No
If you are not sick, would you still wear gloves and/or face masks when going to public places?	◯ Yes ◯ No
Do you think use of gloves and/or face masks by the general population prevent the spread of COVID-19?	◯ Yes ◯ No
If soap and water are not readily available, do you use a hand sanitizer that contains at least 60% alcohol?	◯ Yes ◯ No
Do you avoid touching your eyes, nose, and mouth with unwashed hands?	◯ Yes ◯ No
Do you avoid close contact with people who are sick?	◯ Yes ◯ No
Do you cover your mouth and nose with a tissue or the inside of your elbow when you cough or sneeze?	◯ Yes ◯ No
Do you throw used tissues in the trash?	◯ Yes ◯ No
Do you immediately wash your hands with soap and water for at least 20 seconds after you have coughed or sneezed? If soap and water are not readily available, do you clean your hands with a hand sanitizer that contains at least 60% alcohol?	◯ Yes ◯ No

Do you clean and disinfect frequently touched surfaces daily? This includes tables, doorknobs, light switches, countertops, handles, desks, phones, keyboards, toilets, faucets, and sinks.

◯ Yes ◯ No

If surfaces are visibly dirty, do you clean them with detergent or soap and water prior to disinfection?

◯ Yes ◯ No

On a scale from 0-10, where 0 is no confidence and 10 is the most confidence, how confident are you that you know what you can do to minimize the likelihood of becoming infected with COVID-19?

Please move the slider up or down to best reflect your opinion.

0 (No Confidence) — 10 (Most Confidence)

(Place a mark on the scale above)

On a scale from 0-10, where 0 is no confidence and 10 is the most confidence, how confident are you that other people are following guidelines to minimize the likelihood of spreading COVID-19?

Please move the slider up or down to best reflect your opinion.

0 (No Confidence) — 10 (Most Confidence)

(Place a mark on the scale above)

Do you believe those aged younger than 40 years-old are contributing to the spread of COVID-19 more than those who are 40 years-old and older?

◯ Yes ◯ No

Page 6/6

Do you believe you spend too much time reading, watching, or listening to news regarding COVID-19?	◯ Yes ◯ No
Do you believe the news media is accurately portraying the severity of COVID-19?	◯ Yes ◯ No
Do you believe the news media is inaccurately portraying COVID-19 as more dangerous than it actually is?	◯ Yes ◯ No
Do you believe the news media is inaccurately portraying COVID-19 as less dangerous than it actually is?	◯ Yes ◯ No
Do you believe someone you know will become infected with COVID-19?	◯ Yes ◯ No
Do you believe you will become infected with COVID-19?	◯ Yes ◯ No
Do you believe someone you know will become seriously ill and/or die due to COVID-19?	◯ Yes ◯ No
Do you believe you will become seriously ill and/or die due to COVID-19?	◯ Yes ◯ No
Do you believe that you need to be tested for COVID-19?	◯ Yes ◯ No

On a scale from 0-10, where 0 is no concern at all, and 10 is the most concern, how concerned are you about the impact of COVID-19 on your personal health?

Please move the slider up or down to best reflect your opinion.

0 (No Concern) 10 (Most Concern)

(Place a mark on the scale above)

On a scale from 0-10, where 0 is no concern at all, and 10 is the most concern, how concerned are you about the impact of COVID-19 on your personal financial stability?

Please move the slider up or down to best reflect your opinion.

0 (No Concern) 10 (Most Concern)

(Place a mark on the scale above)

On a scale from 0-10, where 0 is no concern at all, and 10 is the most concern, how concerned are you about the negative impact of COVID-19 on the overall US economy?

Please move the slider up or down to best reflect your opinion.

0 (No Concern) 10 (Most Concern)

(Place a mark on the scale above)

On a scale from 0-10, where 0 is no concern at all, and 10 is the most concern, how concerned are you about the negative impact of COVID-19 on the overall global economy?

Please move the slider up or down to best reflect your opinion.

0 (No Concern) 10 (Most Concern)

(Place a mark on the scale above)

Please rate on a scale from 0-10 how appropriately you believe the US government and CDC have responded to the COVID-19 pandemic, where 0 indicates that you do not think the government and CDC have responded appropriately at all, and 10 indicates that you think the government and CDC have responded perfectly.

Please move the slider up or down to best reflect your opinion.

0 (Inappropriate Response) 10 (Perfect Response)

(Place a mark on the scale above)

On a scale from 0-10, where 0 is not severe at all, and 10 is the most severe, how severe do you believe the COVID-19 outbreak to be? Please consider the following information from the CDC (published March 16, 2020), as well as your own perceptions of the severity. The case mortality rate has been estimated to be 3.5% in China (as of March 16, 2020).

Please move the slider up or down to best reflect your opinion.

0 (Not Severe) 10 (Most Severe)

(Place a mark on the scale above)

Have you stocked up on supplies? ◯ Yes ◯ No

Do you take precautions everyday to keep space between yourself and others? ◯ Yes ◯ No

---

When you go out in public, do you keep away from others who are sick, limit close contact and wash your hands often? ◯ Yes ◯ No

---

Are you avoiding crowds as much as possible? ◯ Yes ◯ No

Are you avoiding cruise travel and non-essential air travel? ◯ Yes ◯ No

---

If there is a COVID-19 outbreak in your community will you plan to stay home as much as possible to further reduce your risk of being exposed? ◯ Yes ◯ No

---

Have you contacted your healthcare provider to ask about obtaining extra necessary medications to have on hand in case there is an outbreak of COVID-19 in your community and you need to stay home for a prolonged period of time? ◯ Yes ◯ No
