# Supplementary figures and images for: The Public’s Perception of the Severity and Global Impact at the Start of the SARS-CoV-2 Pandemic: A Crowdsourcing-Based Cross-Sectional Analysis
Source: J Med Internet Res. 2020 Nov 26;22(11):e19768. doi: 10.2196/19768 (PMC7695545; doi:10.2196/19768)

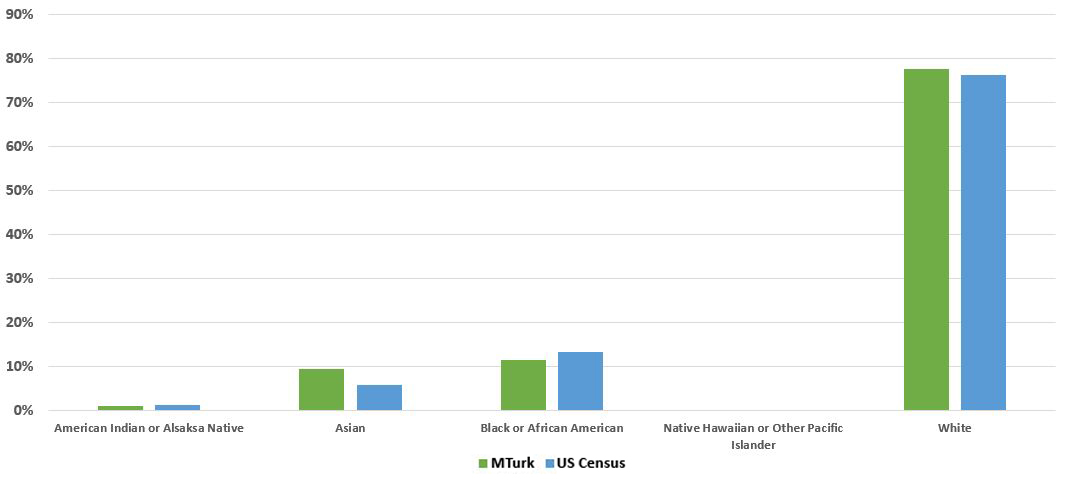

Supplement: Multimedia Appendix 3 [file jmir_v22i11e19768_app3.png]

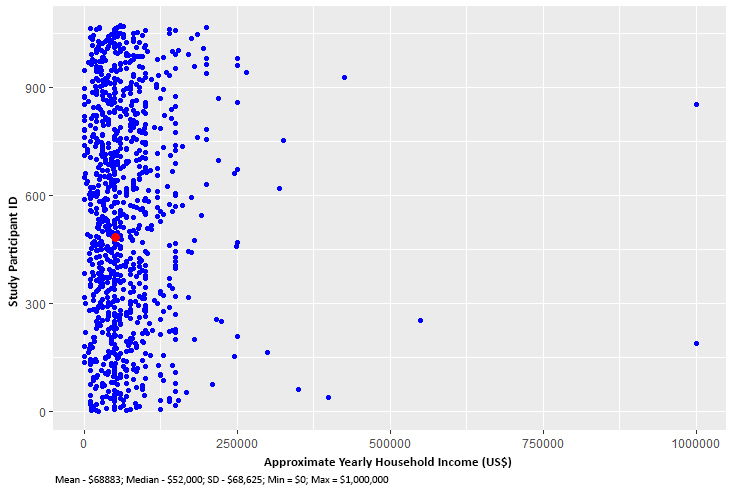

Supplement: Multimedia Appendix 4 [file jmir_v22i11e19768_app4.png]

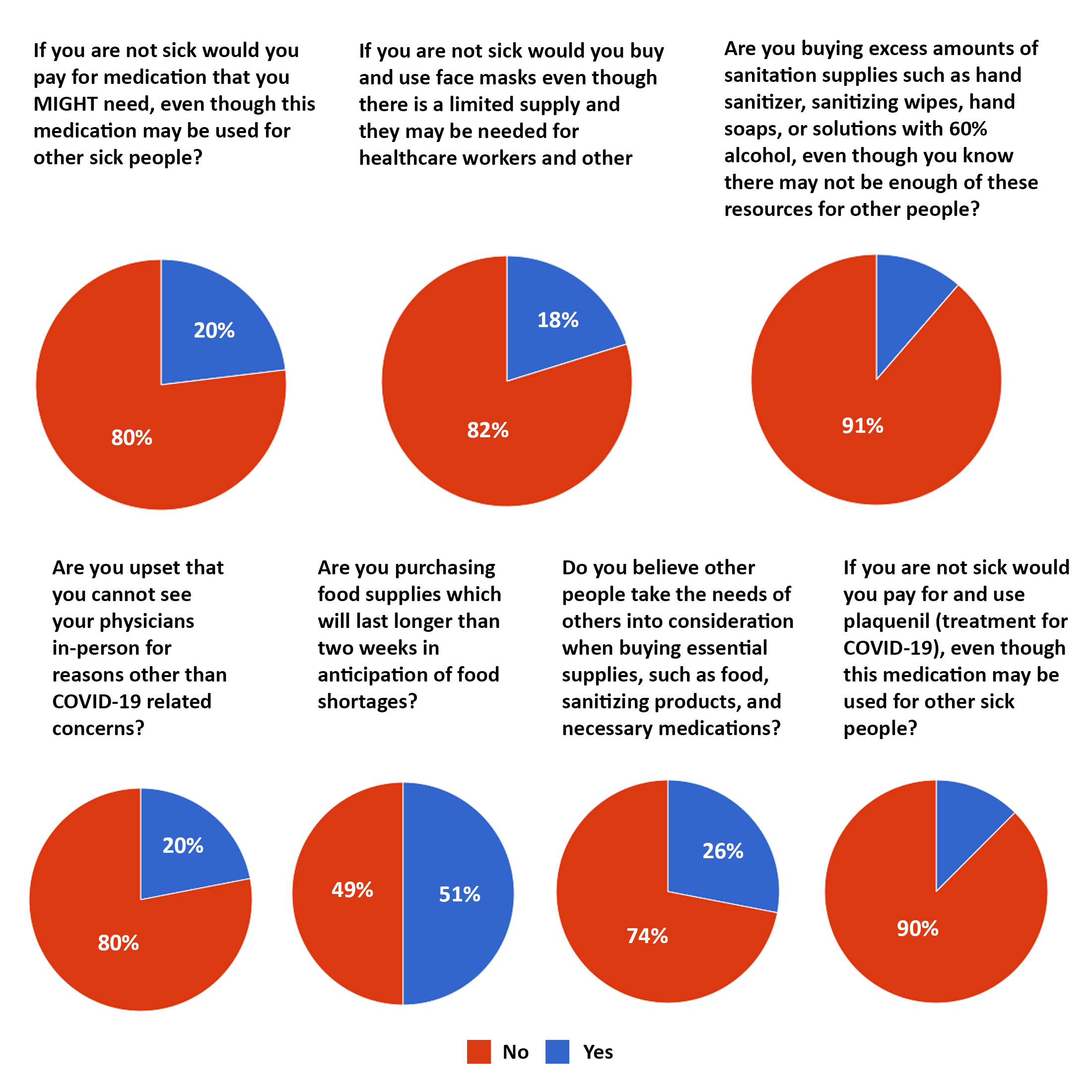

Supplement: Multimedia Appendix 5 [file jmir_v22i11e19768_app5.png]

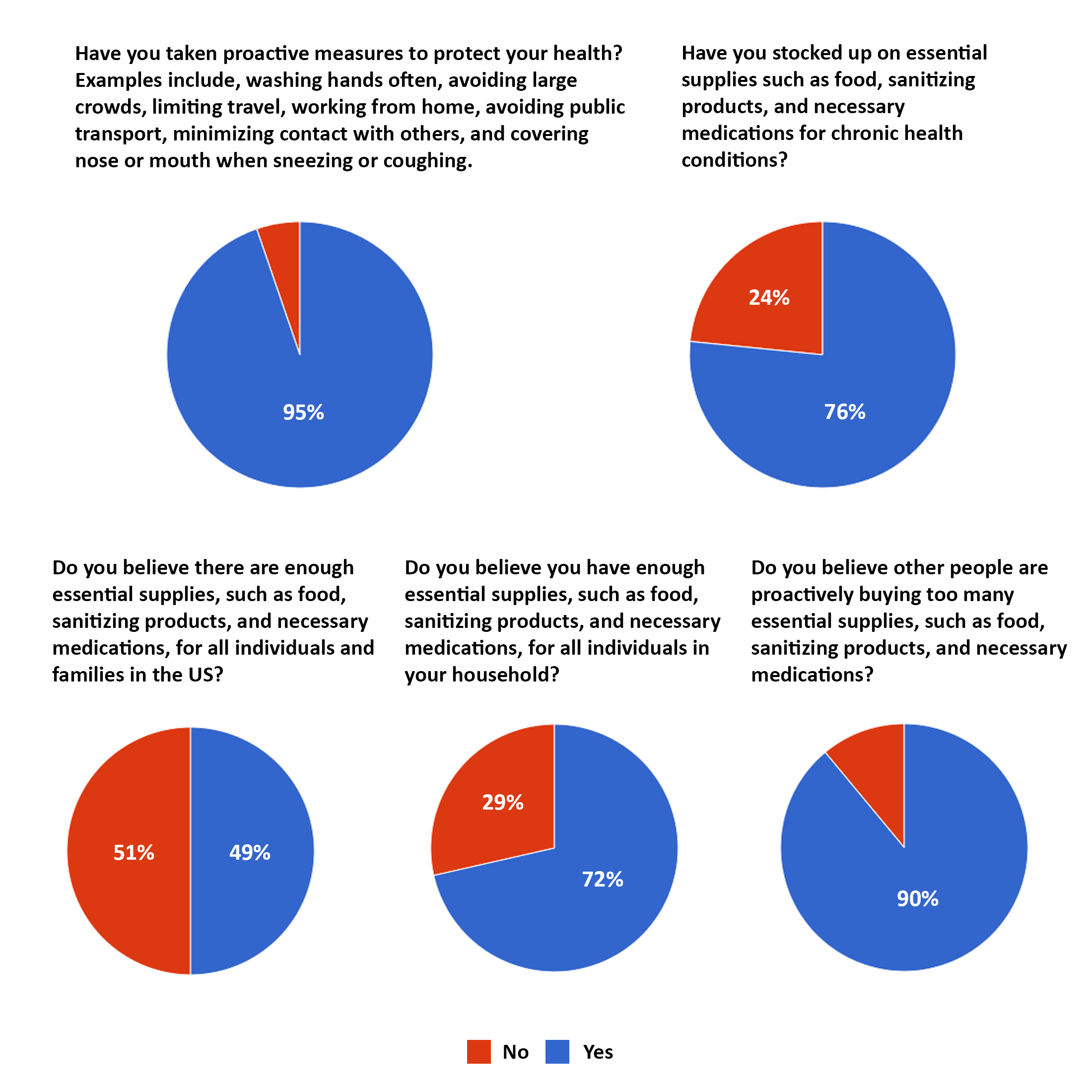

Supplement: Multimedia Appendix 6 [file jmir_v22i11e19768_app6.png]
